# Live birth following donor oocyte IVF/ICSI with surplus cryopreserved MicroTESE retrieved sperm: a case report

**DOI:** 10.1007/s10815-014-0421-y

**Published:** 2015-01-13

**Authors:** Parviz K. Kavoussi, Kate C. Odenwald, Roxanne B. Summers-Colquitt, Jason E. Swain, Thomas B. Pool, Shahryar K. Kavoussi

**Affiliations:** Austin Fertility & Reproductive Medicine/Westlake IVF, 300 Beardsley Lane, Building B, Suite 200, Austin, TX 78746 USA

## Introduction

Until 1999, men with non-obstructive azoospermia (NOA) did not have an option to conceive using their sperm [1]. Since the advent of microdissection testicular sperm extraction (MicroTESE), men with NOA have a chance to conceive by using their sperm for in vitro fertilization/intracytoplasmic sperm injection (IVF/ICSI). With more widespread use of serum antimüllerian hormone (AMH) testing to assist in the evaluation of diminished ovarian reserve (DOR), the controversy of appropriate options for couples with both of these obstacles has arisen.

## Materials and methods

This case is reported after chart review of a successful outcome.

## Results

A 47 year old man with NOA and his 42 year old wife with DOR were evaluated for subfertility in a couples fertility center by a Reproductive Endocrinologist (SKK) and a Reproductive Urologist (PKK). The male partner was found to have 9 cc volume testes on physical examination and did not have a palpable varicocele. He was found to be azoospermic on two semen analyses with normal semen volumes on both. His gonadotropins were normal with an FSH level of 4.1 mIU/ml and an LH level of 3.2 mIU/ml; his prolactin level was 9.8 ng/ml. He was eugonadal with a testosterone of 498 ng/dl and an estradiol of 27 pg/ml; therefore, medical treatment was not indicated prior to MicroTESE. His karyotype revealed a normal 46XY result and Y chromosome microdeletion assay revealed no microdeletions in the AZFa, AZFb, or AZFc regions.

They were counseled extensively regarding advanced maternal age, and elected to proceed with fresh MicroTESE and IVF/ICSI with the female partner’s own oocytes. The female partner underwent 12 days of controlled ovarian stimulation (COS) with 225 IU of recombinant FSH and 75 IU of human menopausal gonadotropins per day, with an estradiol level of 1143 pg/ml, progesterone level of 0.5 ng/ml, and a trilaminar appearing endometrium with a thickness of 11.0 mm on the day of hCG trigger. Sperm were retrieved with unilateral MicroTESE, sufficient in quantity for the fresh IVF/ICSI case as well as for surplus sperm cryopreservation into one vial. Fresh MicroTESE sperm was used for ICSI with five metaphase II oocytes (one out of 6 oocytes retrieved was a germinal vesicle). Two of the mature oocytes fertilized normally into 2PN zygotes, two others had supernumerary nuclei, and one did not fertilize. The two pronuclear zygotes underwent cleavage and, in accordance with the Society for Assisted Reproductive Technology (SART) embryo grading system, [2] were graded as 5A and 6B on day 3 when embryo transfer was performed. The patient unfortunately had a negative pregnancy test. A small portion of testicular tissue was obtained at the time of MicroTESE in order to minimize tissue damage and preserve hormonal testicular function. Histopathology revealed a hypospermatogenesis architectural pattern only.

For their next attempt, when the wife was 43 years of age with an AMH level of <0.16 ng/ml, they elected to use his surplus cryopreserved sperm with donor oocyte IVF/ICSI. The wife’s endometrial preparation with an estradiol and progesterone protocol resulted in a trilaminar endometrium with a thickness of 15.5 mm. The couple’s oocyte donor underwent 8 days of COS with 225 IU of recombinant FSH and 75 IU of human menopausal gonadotropins per day, with an estradiol level of 2266 pg/ml and progesterone level of 0.8 ng/ml on the day of hCG trigger. Twenty-seven oocytes were retrieved, of which 17 were metaphase II oocytes. One vial of MicroTESE sperm was thawed, which yielded a pre-wash, post-thaw sperm concentration of 1.8 million/ml with 0% motility in a volume of 1.0 ml. Sperm preparation and pentoxifylline treatment yielded a post-wash sperm concentration of 1.0 million/ml and 30% motility, 10% progressive motility, in a volume of 0.5 ml. ICSI was performed for the 17 mature oocytes, of which 11 fertilized and yielded 2PN zygotes and 2 yielded a 1PN embryo; four mature oocytes did not fertilize. Two day 3 embryos, graded 8A and 8B, in accordance with the SART embryo grading system [2], were used for embryo transfer and resulted in pregnancy and live birth of healthy dizyogotic twins. In addition, two surplus blastocysts, each graded as 3BB in accordance with the Gardner embryo grading system, were cryopreserved [3, 4].

## Discussion

One percent of men in the general population are azoospermic, and 15% of men presenting for a subfertility evaluation will be found to be azoospermic [5]. In men with NOA, MicroTESE should be offered by Reproductive Urologists with an expertise in this operation to provide the highest success rates at retrieval. As testing for DOR advances, couples may be counseled more appropriately. There is no more challenging of a scenario in couples fertility care, than when the male partner has NOA and the female partner has significant DOR. This case demonstrates that couples in this situation should be offered MicroTESE after an appropriate evaluation in efforts to use the male partner’s sperm. Published successful sperm retrieval rates with MicroTESE range up to 70% depending on the pathology, and rates are highly surgeon dependent [6–11]. This case proves that surplus MicroTESE retrieved sperm can be cryopreserved and used in conjunction with IVF/ICSI with donor oocytes for female partners with DOR to successfully allow the couple to conceive. The advantages of cryopreserving surplus MicroTESE retrieved sperm obtained prior to COS for IVF/ICSI has been evaluated [12] and applies to donor oocyte IVF/ICSI as well. As our understanding of couples fertility continues to advance, we should continue to offer couples all of their options, even in highly challenging circumstances.

## Conclusions

To our knowledge, this is the first case reported of live birth with the use of surplus cryopreserved sperm retrieved from a MicroTESE with donor oocyte IVF/ICSI. In couples with non-obstructive azoospermia and diminished ovarian reserve, MicroTESE should be offered and surplus retrieved sperm may be cryopreserved to be used for ICSI with donor oocytes with a cycle subsequent to sperm retrieval. DOR should not be a deterrent to offering microTESE to these men with NOA. The couple can be successful with IVF/ICSI with surplus cryopreserved sperm retrieved at microTESE and donor oocytes.
